# Acute endophthalmitis following 23-gauge sutureless transconjunctival vitrectomy

**DOI:** 10.4103/0301-4738.77048

**Published:** 2011

**Authors:** Osman Çekiç, Mehmet Çakir, Serpil Yazgan, Ö Faruk Yilmaz

**Affiliations:** Department of Ophthalmology, Beyoğlu Eye Training and Research Hospital, İstanbul, Turkey

**Keywords:** 23-Gauge sutureless vitrectomy, endophthalmitis, epimacular membrane, pars plana vitrectomy

## Abstract

We report a case that developed acute postoperative endophthalmitis after transconjunctival sutureless vitrectomy using the 23-gauge system. A 66-year-old man underwent non-sutured 23-gauge pars plana vitrectomy for epimacular membrane. Since the patient developed signs of acute endophthalmitis and decreased visual acuity to counting fingers on the second postoperative day, re-vitrectomy with silicone oil was performed. The patient responded well to re-vitrectomy, injection of silicone oil and intravitreal antibiotic injections. Methicillin resistant Staphylococcus epidermidis was cultured from vitreous samples. Silicone oil was extracted at 11 months. The patient remains stable at 14 months with a final visual acuity of 20/50.

The transconjunctival sutureless 23-gauge vitrectomy system was first introduced by Eckardt in 2005.[1] Both 23-gauge and 25-gauge sutureless vitrectomy approach have been found as reliable and efficient as 20-gauge system in selected cases. Rapid postoperative healing is observed presumably due to decreased operating time and surgical manipulation, and absence of inflammatory conjunctival reaction related to suture absorption.[1–4] However, transconjunctival sutureless vitrectomy is not totally devoid of complications such as endophthalmitis (postoperative endophthalmitis linked to transconjunctival 25-gauge vitrectomy has been reported).[5,6] Significantly increased incidence of endophthalmitis after 25-gauge vitrectomy has been found (0.23%) compared to that after 20-gauge vitrectomy (0.018%),[6] whereas no increased risk of endophthalmitis following sutureless vitrectomy has been reported in a recent large series that compares 20-gauge and 23-gauge pars plana vitrectomy (0.03% versus none).[7] Here we present a case with acute bacterial endophthalmitis that developed following 23-gauge vitrectomy for epimacular membrane removal.

## Case Report

A 66-year-old man presented with gradual loss of vision of the left eye over 4 years. Visual acuity was 20/20 in the right eye and 20/40 in the left eye. Anterior segment examination was within normal limits except for slight cortical opacity bilaterally. Intraocular pressures were 14 mmHg in the right eye and 15 mmHg in the left eye. Fundus examination revealed a dense epimacular membrane in the left eye. Fundus examination in the right eye was non-specific. The patient underwent epiretinal membrane removal using 23-gauge transconjunctival sutureless pars plana vitrectomy system. At the beginning of the operation, the eye, including periorbital skin and lashes, was treated with povidone-iodine for 2 minutes. No prophylactic antibiotic was given preoperatively. Transconjunctival approach was performed as follows. The conjunctiva was moved laterally from the sclerotomy site with forceps, and a two-step oblique scleral tunnel entry was accomplished with the trocars. The operation included triamcinolone acetonide assisted intentional posterior vitreous detachment and complete removal of posterior hyaloid and epiretinal membrane whilst incomplete removal of peripheral vitreous. At the end of the procedure, partial fluid–air exchange was made. Then, the 23-gauge cannulas were extruded and each sclerotomy site was massaged with a Q-tip, immediately after removing each cannula. The eye was left digitally normotensive. There was no apparent vitreous prolapse from the sclerotomy sites at the conclusion of the vitrectomy. Following the operation, the patient was put on topical preparation of lomefloxacin five times in a day. On the postoperative first day, visual acuity was 20/50, and no signs of endophthalmitis in anterior or posterior chambers were revealed. Intraocular pressure was 11 mmHg. The patient presented with a complaint of intractable pain as well as decreased vision in the operated eye on the second postoperative day. Examination revealed a visual acuity of counting fingers, deep conjunctival injection, and 2 mm hypopyon in the anterior chamber. Intravitreal cavity had massive condensations interfering with fundus examination. B-scan ultrasonography was consistent with endophthalmitis. Intravenous application of 1 g vancomycin 12 hourly and 2 mg ceftazidime 8 hourly as well as hourly topical instillation of vancomycin (25 mg/ml) and ceftazidime (50mg/ml) were started immediately. The patient was also injected intravitreal vancomycin (1.0 mg/0.1 ml) and ceftazidime (2.0 mg/0.1 ml). On the third day, the patient underwent phacoemulsification with intraocular lens implantation, and complete removal of peripheral vitreous condensations with 23-gauge pars plana vitrectomy and silicone oil injection. Intravitreal vancomycin (1.0 mg/0.1 ml) and ceftazidime (2.0 mg/0.1 ml) were also injected at the conclusion of the vitrectomy.

**Figure 1:**
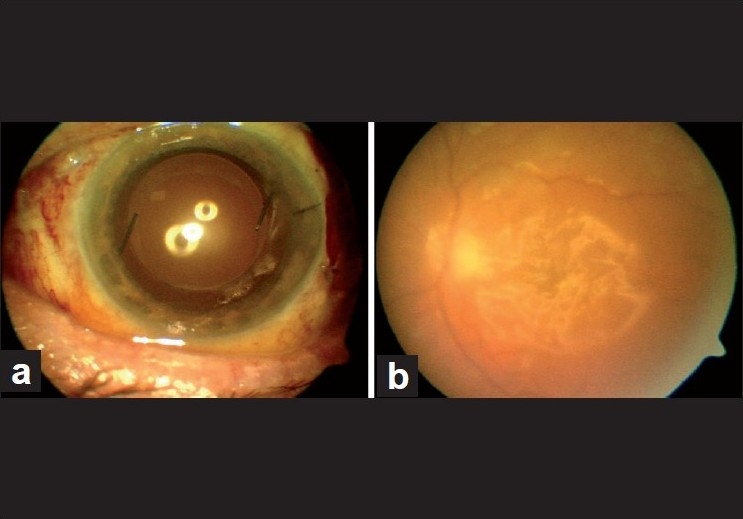
Anterior (a) and posterior (b) chamber pictures of the patient following re-vitrectomy

**Figure 2:**
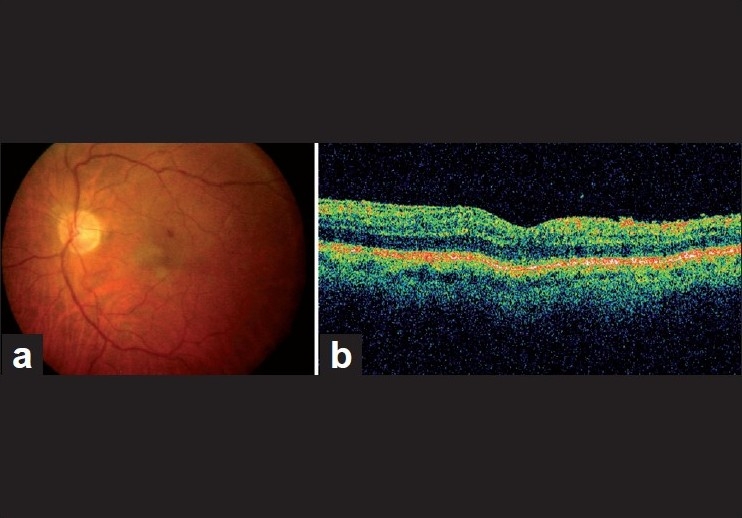
Color fundus picture (a) and optical coherence tomography imaging (b) of the patient after silicone oil removal

Vitreous cultures were positive for methicillin resistant *Staphylococcus epidermidis*. He was treated with systemic moxifloxacin, vancomycin and ceftazidime, and topical fortified vancomycin, ceftazidime and prednisolone acetate instilled every hour postoperatively. Intraocular inflammation subsided within 3 days after vitrectomy [Fig. 1]. At the postoperative second week, visual acuity improved to 20/40. Topical drops were gradually decreased and stopped over a month. At 6 months postoperatively, visual acuity was 20/100 as posterior capsular opacity developed. At 11 months, the silicone oil was removed, and further peeling of epiretinal membrane and surgical posterior capsulotomy was performed. The final vision was 20/50 and the retina was attached at the 14th postoperative month [Fig. 2].

## Discussion

The 23-gauge system is a current popular method for vitreoretinal surgery. Pars plana vitrectomy using 23-gauge is very comfortable for both the surgeon and the patient.[8] Rates of sclerotomy leakage, hypotony, and choroidal detachment were favorable compared with or lower than previously published rates in 25-gauge systems.[3,9] Endophthalmitis is very rare, but a serious issue following vitrectomy.[10] The development of acute endophthalmitis following 25-gauge transconjunctival sutureless vitrectomy has recently been reported.[5] Although it has not been reported yet, the same concern is also valid theoretically for the non-sutured 23-gauge vitrectomy approach despite the oblique scleral entry and conjunctival coverage of the port.

Acute postoperative endophthalmitis can be seen following 23-gauge sutureless vitrectomy as in this case. In our patient, entry of microorganism through sutureless incision site, most probably via prolapsed peripheral vitreous, might be speculated as the peripheral vitreous was left at the first operation. Early postoperative hypotony has been thought to be a possible risk factor for postoperative endophthalmitis after sutureless vitrectomy.[6] To prevent the infection, Parolini *et al*.[7] suggested paying particular attention to the following steps during sutureless 23-gauge surgery: disinfection of the conjunctival fornix with povidone-iodine for at least 1 minute before stating operation, displacement of conjunctiva to mismatch the entry sites into conjunctiva and sclera, proper tunnel shape sclerotomy construction, complete central and peripheral vitrectomy with scleral depression, stopping the infusion when an instrument is extruded from the eye or use a valve system for the cannulas, meticulous closure of the wound by massaging with a Q-tip and with forceps immediately after removing the trocars, careful checking for wound leakage both before removing the infusion cannula at 20 mmHg and after removing the infusion cannula and injection of an air bubble in the presence of any wound leakage.

Our patient responded well to 23-gauge re-vitrectomy, removal of anterior and posterior condensations, and injection of silicone oil, intravitreal antibiotic injections as well as appropriate topical and systemic therapy. To clean vitreous base completely and to overcome preexisting cortical opacity, vitrectomy was also combined with phacoemulsification and intraocular lens implantation. No signs of retinal toxicity due to standard dose of intravitreal drugs in silicone filled eye were observed contrary to[11] a previous report. An acceptable level of visual acuity could also be rescued.
